# Radiation exposure of the spleen during ^177^Lu-DOTATATE treatment and its correlation with haematological toxicity and spleen volume

**DOI:** 10.1186/s40658-016-0153-4

**Published:** 2016-08-05

**Authors:** Johanna Svensson, Linn Hagmarker, Tobias Magnander, Bo Wängberg, Peter Bernhardt

**Affiliations:** 1 Department of Oncology, Sahlgrenska University Hospital, 41345 Gothenburg, Sweden; 2 Department of Radiation Physics, Sahlgrenska University Hospital, Gothenburg, Sweden; 3 Department of Surgery, Institute of Clinical Sciences, The Sahlgrenska Academy, Sahlgrenska University Hospital, Gothenburg, Sweden; 4 Department of Medical Physics and Biomedical Engineering, Sahlgrenska University Hospital, Gothenburg, Sweden

**Keywords:** PRRT, ^177^Lu-DOTATATE, Spleen dosimetry, Haematological toxicity

## Abstract

**Background:**

Somatostatin analogue-based radionuclide therapy with ^177^Lu-DOTATATE is an important treatment option for patients with advanced neuroendocrine tumours overexpressing somatostatin receptors. In addition to the kidneys, the bone marrow is a major dose-limiting organ. The correlation between developed haematological toxicity and absorbed dose to the bone marrow is poor, which indicates that other factors affect haematological response. The spleen has an important role in the haematopoetic system, including being a reservoir for blood cells. It is also the organ that receives the highest mean absorbed dose during ^177^Lu-DOTATATE treatment. The aim of this study was to analyse mean absorbed dose to the spleen and its correlation with haematological toxicity, and to explore changes in splenic volume.

The study included 41 patients treated with 7.2 GBq (3.5–8.3 GBq) of ^177^Lu-DOTATATE on two to five occasions. Following each fraction, planar whole-body scans were acquired at 2, 24, 48, and 168 h, and a SPECT/CT at 24 h post-injection. Mean absorbed spleen dose was calculated utilising planar images for time-activity data and SPECT to adjust activity amounts. Splenic volume information was collected from diagnostic CT scans at baseline and follow-up.

**Results:**

Median and total absorbed spleen doses were estimated to 4.5 and 15 Gy, respectively. Total absorbed spleen dose correlated with decrease in Hb (*p* = 0.02), but not WBC (*p* = 0.31) or PLT (*p* = 0.65) counts. For patients without bone metastases, mean absorbed spleen dose correlated with decrease in PLT (*p* = 0.04) but not Hb (*p* = 0.16) or WBC (*p* = 0.42) counts. The spleen volume was reduced to 75 % (*p* < 0.001) of original values (200 vs. 260 ml) at a mean follow-up of 36 months.

**Conclusions:**

Haematological toxicity according to Hb counts was moderately but significantly correlated with total absorbed spleen dose. This supports the possibility that radiation exposure of the spleen affects overall haematological response during ^177^Lu-DOTATATE treatment.

## Background

Somatostatin analogue-based PRRT using ^177^Lu-DOTATATE for patients with advanced neuroendocrine tumours that overexpress somatostatin receptors has become an established treatment option included in published guidelines [1]. A majority of treated patients are reported to have clinically relevant effects in terms of symptom relief, improved quality of life, and radiological response [2–4]. The dose-limiting organs for all PRRT are mainly the kidneys and the bone marrow [2, 3]. Mean absorbed dose to the kidneys is routinely calculated at each fraction, and treatment is usually ended at a fixed absorbed dose to avoid the risk of serious renal complications, as a possible dose-response relationship between kidney dose and renal function has been described [5, 6]. However, clinically relevant renal toxicity is very seldom reported in ^177^Lu-based PRRT [2, 7].

Haematological toxicity, used as an indirect measure of bone marrow exposure, more often occurs during treatment, and serious cytopenia (grade 3 or 4 according to the National Cancer Institute’s Common Terminology Criteria for Adverse Events, version 3.0) has been described in 10 % of patients in larger reports [2, 8]. However, individual bone marrow dosimetry is only occasionally performed during ^177^Lu-DOTATATE treatment, both because of the lack of a clear dose-response relationship and the assumption that the dose to the bone marrow will not exceed the used dose limit of 2 Gy [2, 9, 10]. The poor correlation between mean absorbed dose to the bone marrow and developed haematological toxicity in published studies [9, 10] is probably due to the fact that several factors influence haematological response. Earlier studies on haematological toxicity during ^177^Lu-DOTATATE treatment have revealed associations with renal function, tumour burden [11, 12], initial cytopenia [8, 11], previous chemotherapy, and patient age [11].

An additional factor that may affect the development of haematological toxicity is radiation exposure of the spleen. In a long-term follow-up study of 203 patients receiving ^177^Lu-DOTATATE, 16 patients with earlier splenectomy developed less haematological toxicity [8]. The spleen is part of the immune system; it has the ability to produce blood cells and acts as a reservoir for red and white blood cells and platelets [13–15]. Furthermore, it is often reported to be the organ that receives the highest mean absorbed dose during PRRT [3, 16]. The reason for the radiation exposure is physiological uptake of somatostatin in the splenic tissue [17, 18]. Immunohistochemical studies combined with measurement of mRNA reveal a predominance of somatostatin receptor subtype 2A in the spleen [19]. This receptor subtype is preferred by both the somatostatin analogues most often used to diagnose and treat neuroendocrine tumours: octreotide and octreotate.

Prior knowledge about radiation exposure of the spleen was obtained from studies of external radiotherapy, where the spleen is irradiated to relieve pain due to splenic enlargement from malignant disease. Side effects from this treatment are described in terms of a decline in haemoglobin (Hb), white blood cell (WBC), and platelet (PLT) counts, which may be due to direct exposure of the blood cells that are physiologically pooled in the spleen, or to a response from haematopoetic cells situated in the spleen [20–24]. Radiation response was also demonstrated in these studies by a desired reduction in the volume of the spleen; as a result of this, effective pain relief is achieved. The aim of this study was to examine the possible role of the spleen in the development of haematological toxicity during ^177^Lu-DOTATATE treatment. Changes in splenic volume at follow-up were also explored.

## Methods

### Patients and treatment characteristics

This study included 41 patients with advanced neuroendocrine tumours. The study was approved by the Regional Ethics Review Board in Gothenburg, Sweden, and performed in accordance with the principles of the Declaration of Helsinki and national regulations. The need for written informed consent was waived. Patients included in the study had tumours judged to overexpress somatostatin receptors (i.e., uptake exceeding physiological liver uptake) by somatostatin receptor scintigraphy (^111^In-DTPA-octreotide, Octreoscan^®^; Mallinckrodt). All patients had clinically, biochemically, or radiologically confirmed progressive disease and a renal ^51^Cr-EDTA clearance >40 mL/min/1.73 m^2^. Each fraction was administered as a 30-min infusion of 7.2 ± 1.3 GBq (3.5–8.3 GBq) of ^177^Lu-DOTATATE coinfused with kidney-protecting amino acids (2.5 % lysine and 2.5 % arginine in 1 L of 0.9 % NaCl, infusion rate 250 mL/h) to a total amount of 24 ± 7.1 GBq (8.2–40 GBq) on two to five occasions, 6–8 weeks apart.

### Dosimetry

For dosimetry of at-risk organs, including the spleen, planar images were acquired at 2, 24, 48, and 168 h post-injection (p.i.), and single photon emission computed tomography (SPECT)/CT was performed at 24 h p.i. To calculate the mean absorbed dose to the spleen, a hybrid technique was used, that utilised information from both planar and SPECT images. The spleen was identified, and a region of interest (ROI) was drawn on the planar images. For background subtraction, a small ROI below the spleen was drawn. The activity in the ROI was calculated for the different time points according to the conjugate view method, as described in a previous study [12]. For this calculation, the thickness of the spleen and the thickness of the body over the spleen were acquired from the 24-h SPECT/CT. A volume of interest (VOI) around the spleen was also drawn from the 24-h SPECT; the activity amount in the VOI was then calculated and the activity concentration decided. A factor of 0.85 was used to correct for the partial volume effect, which was determined from SPECT simulation studies using the research image platform PhONSAi, developed in-house [25]. The accuracy of the SPECT simulation of the partial volume effect was verified against measurement of the partial volume effect in the NEMA phantom, and a high agreement was found. The acquired activity concentration from the SPECT was applied to adjust the planar time-activity curves, and the accumulated activity within the spleen was calculated from the area under the curve. The absorbed dose to the spleen could then be calculated from the equation:

$$ {D}_{\mathrm{spleen}}=\frac{\tilde{A}\cdot \varDelta \cdot \varphi }{M}, $$

where *Ã* is the adjusted accumulated activity in the spleen, Δ is the energy emitted by the radionuclide per disintegration (147 keV for ^177^Lu, [26]), *φ* is the absorbed fraction of energy from the β-particles, which was assumed to be one, and *M* is the mass of the spleen with a supposed density of unity. The mean absorbed dose was estimated by assuming local absorption of the β-particles emitted from ^177^Lu and neglecting the minor contribution from photons (≤2 %; [27, 28]).

Diagnostic CT scans were used to estimate splenic volumes before treatment and in long-term follow-up by manual ROI drawing in all CT slices in the image platform PhONSAi. All diagnostic CT scans used for spleen volume estimates were contrast-enhanced, slice thickness 5 mm.

Bone marrow dosimetry was not performed in this study. This is subject of a separate work by our research group, including development of a novel image-based method for bone marrow dose estimates.

### Assessment of haematological response

Patients were monitored according to bone marrow function (Hb, WBC, and PLT counts) by blood sampling every 2 weeks during the treatment period. Blood samples were analysed according to standard procedures at the hospital. For an estimation of the decline in Hb, WBC, and PLT counts, the nadir value after treatment start was compared to baseline, and this relative value was related to the mean absorbed dose to the spleen per treatment and to the total mean absorbed dose.

### Statistical methods

Associations between a decrease in blood counts from baseline values and the mean absorbed dose to the spleen were investigated by analysis of variance (ANOVA) in linear regression analysis. Residuals of the linear regression analysis (Hb and PLT values) were found to be normally distributed according to the Anderson-Darling test. Means and standard deviation are used to report normally distributed continuous variables, otherwise median values and range are reported. *p* values <0.05 were considered significant.

## Results

**Fig. 1:**
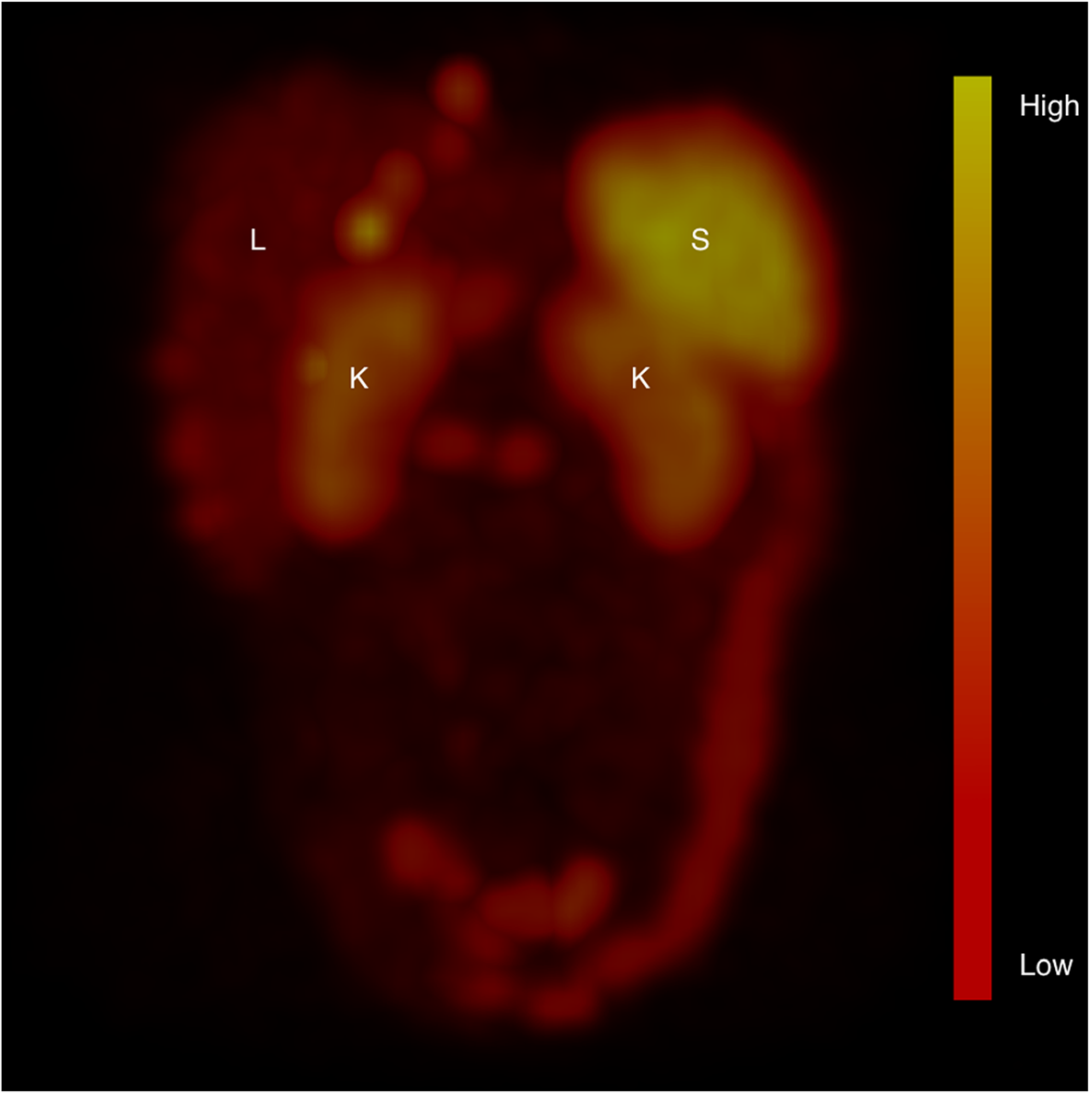
SPECT/CT (acquired 24 h p.i.) of ^177^Lu-DOTATATE illustrating the relatively high physiological uptake of ^177^Lu-DOTATATE in the spleen (*S*) and kidneys (*K*), resulting in high absorbed doses. This patient, a woman with an ileal NET, received a mean absorbed dose of 6.4 Gy to the spleen and 3.1 Gy to the kidneys from this treatment. The *colour bar* indicates the uptake intensity. Multiple tumours with a pathological uptake are present in the liver (*L*)

**Fig. 2:**
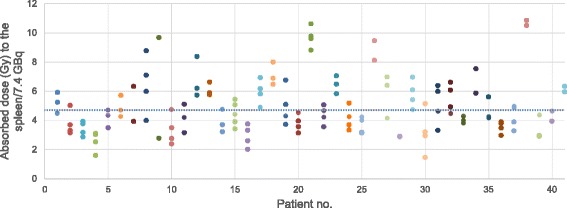
Mean absorbed doses to the spleen per 7.4 GBq of ^177^Lu-DOTATATE for the different fractions. The *grey dotted line* indicates the median absorbed dose per 7.4 GBq for all 41 patients (4.7 Gy; coefficient of variation = 0.19). *No.* number

**Fig. 3:**
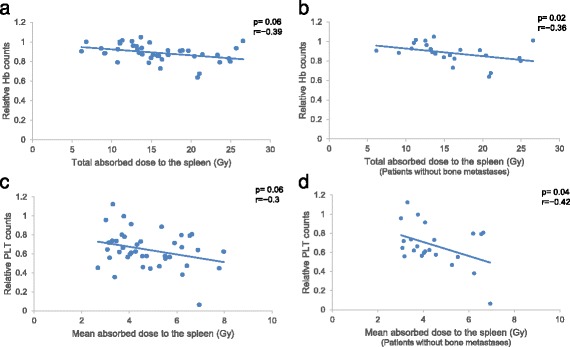
Correlations between total absorbed spleen dose and relative decline in Hb count during ^177^Lu-DOTATATE treatment, for all patients (**a**) and for patients without bone metastases (**b**), and correlations between mean absorbed spleen dose per treatment and PLT count, for all patients (**c**) and for patients without bone metastases (**d**)

As illustrated in Fig. 1, the uptake of ^177^Lu-DOTATATE by the spleen was often higher than uptake by other organs, which resulted in a relatively high mean absorbed spleen dose per treatment for the 41 patients included in the study. Median absorbed dose was estimated to 4.7 Gy (1.5–10.6 Gy) per 7.4 GBq of ^177^Lu-DOTATATE (Fig. 2). For all treatments, the median absorbed spleen dose was estimated to 15 Gy (5.8–39 Gy) from an average activity amount of 24 ± 7.1 GBq (8.2–37 GBq). A moderate correlation was seen between total absorbed dose to the spleen and decrease in Hb (*p* = 0.02; Fig. 3) but not WBC (*p* = 0.31) or PLT (*p* = 0.65) counts. For patients without bone metastases (*n* = 26) the correlation with Hb counts was not significant (*p* = 0.06; Fig. 3). Mean absorbed dose to the spleen per treatment seemed associated with a decrease in PLT counts, although not significantly (*p* = 0.06). For patients without bone metastases, it reached significance (*p* = 0.04; Fig. 3). No statistically significant correlation was obtained between mean absorbed bone marrow dose and Hb (*p* = 0.16) or WBC (*p* = 0.42) counts.

**Fig. 4:**
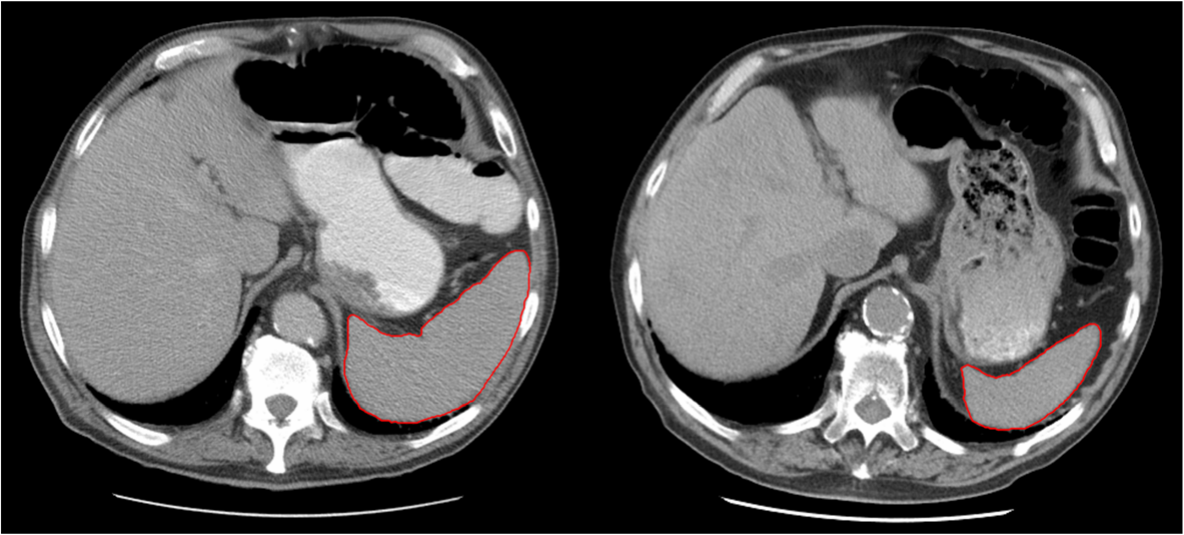
Patient with an ileal NET that metastasised to abdominal lymph nodes. The patient received three fractions of ^177^Lu-DOTATATE to a total amount of 22 GBq. Splenic volume at baseline was 415 cm^3^ (*left*). At follow-up 5 years after treatment start, splenic volume was 167 cm^3^ (*right*), a volume reduction of 60 %. Total absorbed dose to the spleen was estimated to 13 Gy

A general decline in splenic volume was observed after ^177^Lu-DOTATATE treatment. Mean volume of the spleen (*n* = 31) was 260 mL (54–640 mL) before treatment start, compared to 200 mL (41–600 mL) after a mean follow-up of 36 months from the first fraction given, which represents a decrease to 75 % (40–131 %) of the original volume (*p* < 0.001). Figure 4 shows an example of a patient who experienced a volume reduction of the spleen to 40 % of the original value after three treatments of 177Lu-DOTATATE and a total absorbed spleen dose of 13 Gy. The magnitude of volume reduction was not correlated to mean absorbed spleen dose per treatment (*p* = 0.53) or total absorbed dose for all treatments (*p* = 0.20).

## Discussion

This study found a modest correlation between total absorbed dose to the spleen and Hb counts in blood (*p* = 0.02). The mean absorbed dose to the spleen seemed associated to the decrease in PLT counts though not significantly (*p* = 0.06). This correlation reached significance for patients with no bone metastases (*p* = 0.04). The reason for these differences may be that patients with bone metastases are a more heterogeneous group including those with one or two bone metastases as well as patients with massive engagement and prerequisites of substantial bone marrow infiltration. The potential influence of the spleen on haematological response was noted also by Sabet et al. [8]. They reported a significantly lower degree of haematological toxicity (according to NCI CTCAE) for patients receiving ^177^Lu-DOTATATE, who had previously undergone splenectomy (16 out of 203 patients). This suggests that these patients’ blood cells were protected from being irradiated in the blood reservoir situated in the spleen. However, another published study that included 53 patients treated with ^177^Lu-DOTATATE did not find a correlation between absorbed dose to the spleen and haematological toxicity. This may be due to the fact that the evaluation in this study was made after only one fraction, and a reported mean absorbed dose to the spleen of 6.3 Gy, which could be too small an exposure for a detectable haematological response [29].

A correlation between irradiation of the spleen and the development of cytopenia was reported for external radiotherapy used to reduce splenic volume in patients with haematological disorders, at fraction doses of 0.2–1 Gy/fraction to a total mean absorbed dose of 2–10 Gy [20, 21]. The low absorbed doses required for a haematological response in patients undergoing external radiotherapy may reflect the fact that patients with haematological disorders have other predisposing factors. For example, these patients are likely to have active haematopoesis in the spleen [30], which means that haematopoetic stem cells will be irradiated, while patients in the present study have mainly mature blood cells pooled in the spleen, with different radiosensitivity. In addition, the absorbed dose rate will be considerably higher from external radiotherapy than from ^177^Lu-based radionuclide therapy. The absorbed dose rates in these studies varied between 0.2 and 1 Gy per fraction, supposedly delivered in minutes, while radionuclide therapy from a low/medium-energy β-emitter such as ^177^Lu would be expected to yield an absorbed dose rate of <0.02 Gy/h [31, 32].

Hb counts were correlated with the total mean absorbed dose to the spleen (*p* = 0.02) but not with the mean absorbed dose per treatment. This might reflect a relatively lower radiosensitivity of red blood cells compared to PLTs, as well as a slower regeneration. The regeneration time for red blood cells is estimated to 120 days, compared to 10 days for platelets [13, 33]. The WBC count did not seem to differ with absorbed spleen dose in this study, which could be due to the fact that WBCs are not pooled in the spleen to the same extent and they have a faster regeneration time [33].

The present study clearly demonstrate that the spleen responds to radiation exposure from ^177^Lu-DOTATATE with a reduction in volume, similar to that observed with external radiotherapy [21, 22], despite differences in fractionation, total doses, and splenic function between these two patient groups. Whether the spleen is affected physiologically by the radiation exposure remains to be investigated.

Although a dose-dependent relationship may exist between the mean absorbed dose to the spleen and the blood cell count, there are several other factors that contribute to the haematological response during ^177^Lu-DOTATATE treatment. Recently, associations were observed between haematological toxicity and renal function [11, 12], initial cytopenia [8], tumour burden [11, 12], and patient age [11]. The fact that several biological parameters influence the haematological response may explain why strong correlations with absorbed bone marrow dose are difficult to find. Further analysis to capture the spleen impact on haematological response in relation to other biological parameters will be subject of an upcoming multivariate and compartment analysis by the research group.

## Conclusions

Haematological toxicity according to haemoglobin counts was moderately but significantly correlated with accumulated mean absorbed dose to the spleen during ^177^Lu-DOTATATE treatment. This supports the possibility that also radiation exposure of the spleen affects overall haematological response during this therapy, which illustrates its complexity.
